# Transverse-plane descriptors and modifiers of the SRS-Lenke-Aubin 3D classification provide complementary and clinically informative 3D features beyond 2D assessment in Lenke 1 AIS curves

**DOI:** 10.1007/s43390-026-01470-3

**Published:** 2026-06-13

**Authors:** Carl-Eric Aubin, Brice Ilharreborde, Lawrence G. Lenke, Delphine Aubin, Takashi Kaito, Christiane Caouette, Virginie Lafage, Justin S. Smith, Jeffrey Mullin, A. Noelle Larson, Peter O. Newton, Michael G. Vitale, Michelle C. Welborn

**Affiliations:** 1 Department of Mechanical Engineering, Polytechnique Montréal, Downtown Station, P.O. Box 6079, Montreal, QC H3C 3A7 Canada; 2 Sainte-Justine University Hospital Center, 3175 Côte Sainte-Catherine Road, Montreal, QC H3T 1C5 Canada; 3 AP-HP Hôpital Universitaire Robert-Debré, 48 Boulevard Sérurier, 75019 Paris, France; 4 Department of Orthopedic Surgery, Columbia University Vagelos College of Physicians and Surgeons, New York, NY USA; 5 NewYork-Presbyterian Och Spine Hospital, New York, NY USA; 6 Division of Orthopaedic Surgery, Osaka University Graduate School of Medicine, Osaka, Japan; 7 Division of Orthopaedic Surgery, Osaka Rosai Hospital, Osaka, Japan; 8 Department of Orthopaedic Surgery, Lenox Hill Hospital, Northwell Health, New York, NY USA; 9 Department of Neurosurgery, University of Virginia School of Medicine, Charlottesville, VA USA; 10 Department of Neurosurgery, Jacobs School of Medicine and Biomedical Sciences, University at Buffalo, The State University of New York, Buffalo, NY USA; 11 Department of Orthopedic Surgery, Mayo Clinic, 200 First Street SW, Rochester, MN 55905 USA; 12 Department of Orthopedics, Rady Children’s Hospital, 3020 Children’s Way, San Diego, CA 92123 USA; 13 NewYork-Presbyterian Morgan Stanley Children’s Hospital, New York, NY USA; 14 Department of Orthopaedics and Rehabilitation, Oregon Health & Science University School of Medicine, Portland, OR USA; 15 Shriners Children’s Portland, Portland, OR USA

**Keywords:** Adolescent idiopathic scoliosis, Thoracic scoliosis, SRS-Lenke-Aubin 3D classification, Transverse-plane descriptors, Apical vertebral rotation, Regional plane of deformation

## Abstract

**Purpose:**

To investigate how the transverse-plane descriptors and modifiers of the SRS-Lenke-Aubin 3D classification—orientation of the regional plane of deformation (ORPD), apical vertebral rotation (AVR), and apical displacement (3D distance from the apex to the end-vertebrae line, DAEVL)—characterize Lenke 1 thoracic curves and complement conventional 2D Lenke modifiers and radiographic measures.

**Methods:**

One hundred sixty-two Lenke 1 adolescent idiopathic scoliosis patients underwent 3D reconstruction from preoperative biplanar radiographs. Associations between transverse-plane descriptors (ORPD, AVR, DAEVL) and standard coronal and sagittal radiographic measures (Cobb angles, thoracic kyphosis [TK], lumbar lordosis [LL]) were analyzed across proximal thoracic (PT), main thoracic (MT), and thoracolumbar/lumbar (TL/L) regions. Relationships between classical Lenke modifiers and transverse-plane modifiers derived from the SRS-Lenke-Aubin 3D classification were evaluated using contingency tables, chi-square tests or Fisher-Freeman-Halton exact tests as appropriate, and Cramér’s V. Pearson correlations and multivariate linear regression were used to assess regional coupling and the independent contributions of coronal and sagittal parameters to ORPD. Subgroup analyses compared Lenke 1A, 1B, and 1C patterns.

**Results:**

Across analyses, substantial inter-patient variability was observed, with wide dispersion of transverse-plane descriptors for similar 2D measurements. Relationships between coronal severity and transverse-plane geometry were region-dependent. ORPD showed no statistically significant association with Cobb magnitude in the PT and MT regions (p > 0.05), whereas a moderate and statistically significant association was observed in the TL/L region (r = 0.4792, p < 0.001). Associations between AVR and Cobb magnitude were weak in PT (r = 0.1688, p = 0.0317) and moderate in both MT (r = 0.4424, p < 0.001) and TL/L (r = 0.4085, p < 0.001). ORPD and AVR were not significantly associated in PT, showed a weak inverse association in MT (r = −0.2177, p = 0.0054), and were moderately associated in compensatory TL/L (r = 0.5342, p < 0.001). TK demonstrated a moderate-to-strong inverse association with MT ORPD (r = −0.6344, p < 0.001), while LL showed no significant association with TL/L ORPD (r = 0.1177, p = 0.1357). DAEVL showed moderate associations with Cobb magnitude across regions and selective coupling with ORPD and AVR. Strong associations were observed between the Lenke lumbar modifier and TL/L ORPD, and between the thoracic sagittal profile modifier and MT ORPD (both p < 0.001), indicating partial overlap but suggesting complementarity between 2D and 3D descriptors. Multivariate regression confirmed region-specific behavior, with MT ORPD primarily influenced by sagittal profile and TL/L ORPD predominantly driven by coronal magnitude. Subgroup analyses revealed distinct 3D signatures across Lenke 1A, 1B, and 1C, but the 1C subgroup should be interpreted as exploratory.

**Conclusion:**

In Lenke 1 thoracic curves, given the weak to moderate associations found, ORPD, AVR, and DAEVL thus provide additional, complementary, region-specific information that is not fully explained by conventional 2D parameters. The predominance of weak-to-moderate associations reflects meaningful independence rather than lack of relationship, reinforcing the value of integrating transverse-plane descriptors to enhance 3D characterization of AIS.

## Introduction

Adolescent idiopathic scoliosis (AIS) is a three-dimensional (3D) spinal deformity in which coronal deviation, sagittal imbalance, and axial rotation coexist and interact in complex ways [1]. Among all curve patterns, Lenke 1 (main thoracic curve without structural lumbar curve) represents the most common configuration and a primary focus of posterior spinal fusion. Their biomechanical behavior is marked by a combination of vertebral rotation, rib cage deformity, sagittal plane alterations, and a regional 3D curve-deformation profile that influence both cosmetic appearance and surgical correction strategies [1].

Thoracic curves possess distinctive 3D features due to the rigidity imposed by the rib cage and the asymmetric rib–vertebrae coupling [1–3]. Early 3D analyses of Lenke 1 curves have already demonstrated the presence of distinct 3D subgroups based on plane orientation and sagittal profile, highlighting that coronal similarity may mask substantial transverse-plane heterogeneity [4]. Corrective surgical maneuvers such as rod derotation, in situ rod bending, posteromedial translation, differential rod contouring, or direct vertebral rotation depend heavily on understanding the underlying transverse-plane morphology [5–7]. The degree of apical vertebral rotation and the regional spinal curvature directly influence the amount of rotational stiffness surgeons encounter during reduction maneuvers, while maintaining or restoring thoracic kyphosis [3].

A particular challenge arises in hypokyphotic thoracic curves, defined by T5-T12 thoracic kyphosis < 10°, and sometimes extended to < 20° based on Lenke’s criteria for curve structurality [8, 9]. Hypokyphosis is clinically relevant because it alters the intrinsic 3D geometry of the trunk, increases rotational prominence and rib–vertebrae interactions—as reflected by higher spinal penetration index values—and may limit the effectiveness of rod derotation techniques [10]. Restoring sagittal alignment is essential to prevent postoperative junctional complications and ensure long-term satisfactory outcomes [11].

Despite extensive reliance on the Lenke classification for surgical planning [8, 9, 12], this system does not explicitly describe transverse-plane morphology, thereby limiting its ability to fully capture the three-dimensional nature of spinal deformity and its implications for surgical interpretation and decision-making. The newly introduced SRS-Lenke-Aubin 3D Classification [13] addresses this gap by integrating transverse-plane descriptors that form the basis of two transverse-plane modifiers applied independently to each spinal region (proximal thoracic [PT], main thoracic [MT], and thoracolumbar/lumbar [TL/L]). The resulting system is structured as a three-tiered, four-modifier framework—curve type + lumbar modifier + thoracic sagittal profile modifier + regional and local transverse-plane modifiers [13]. Within this framework, the orientation of the regional plane of deformation (ORPD) and the apical vertebral rotation (AVR) constitute the two classifier-based transverse-plane modifiers, whereas the 3D distance from the apex to the end-vertebrae line (DAEVL) serves as a complementary, non-classificatory spatial descriptor that refines the 3D characterization of apical deviation. Although these descriptors have been examined in a full AIS cohort [13, 14], their relationships and functional behavior may differ meaningfully across specific Lenke curve subtypes.

The present study therefore focuses exclusively on Lenke 1 curves, aiming to determine how these transverse-plane descriptors behave and vary within this subtype and how they complement conventional 2D radiographic measures. More specifically, this study evaluates regional coupling between coronal, sagittal, and transverse-plane parameters; the overlap between conventional Lenke modifiers and ORPD-based modifiers; and subtype-specific 3D signatures across Lenke 1A, 1B, and 1C curves. Limited coupling or low overlap between 2D and 3D descriptors would be interpreted as evidence of complementarity rather than redundancy. This targeted analysis provides deeper insight into the 3D nature of thoracic deformities and lays the groundwork for future investigations into their potential relevance for surgical interpretation.

## Methods

This retrospective analysis included 162 Lenke 1 AIS patients, extracted from a larger cohort of 381 surgical cases reconstructed in 3D from standardized preoperative biplanar radiographs [14]. Inclusion criteria required the availability of high-quality biplanar radiographs enabling reliable 3D reconstruction, while cases with incomplete imaging data or reconstruction constraints were excluded. Imaging data was obtained in clinical or research contexts with appropriate informed consent, and all procedures were conducted under approval from the institutional research ethics board of the lead institution. The cohort comprised 104 Lenke 1A, 35 Lenke 1B, and 23 Lenke 1C curves, based on the conventional lumbar modifier. The process of cohort construction and subgroup classification was designed to ensure consistency between imaging-derived descriptors and Lenke classification criteria. The mean age was 15.1 years old (9.3–21.3) and there were 140 females and 22 males.

**Fig. 1:**
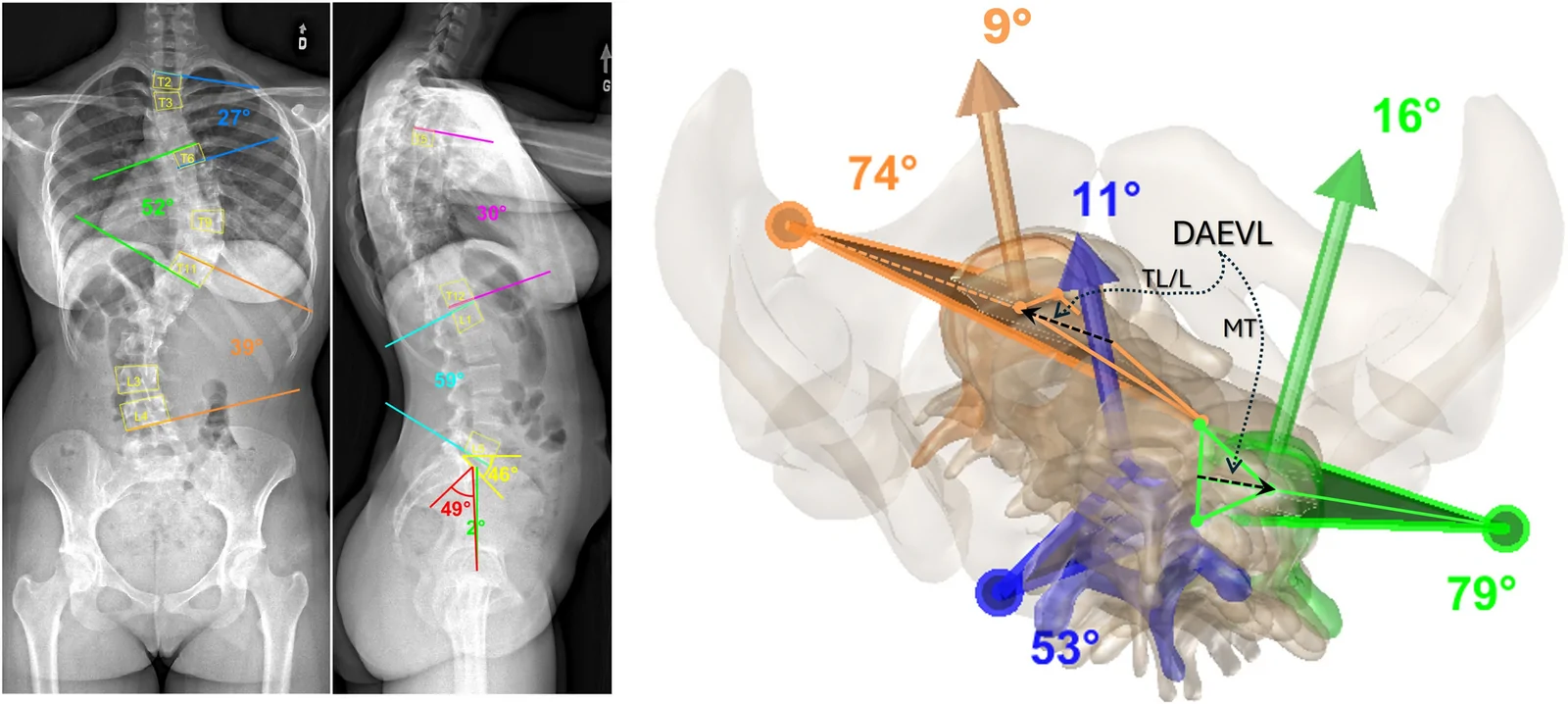
Representative case illustrating standard 2D radiographic measurements—segmental Cobb angles (PT, MT, TL/L), T5–T12 thoracic kyphosis, and L1–L5 lumbar lordosis (left)—and the corresponding top-view representations of the transverse-plane descriptors ORPD, AVR, and DAEVL for the same regions (right). DAEVL corresponds to the shortest perpendicular distance from the apical vertebra to the line joining the end vertebrae of each region

3D reconstructions were performed using validated self-calibrated biplanar algorithms, with previously reported angular accuracy of 2–5° [15–18]. Anatomical landmarks were digitized using established workflows to ensure consistency in the computation of ORPD, AVR, and DAEVL (Fig. 1). Conventional 2D radiographic parameters were obtained from the same imaging set, including segmental (PT, MT, TL/L) Cobb angles, T5-T12 thoracic kyphosis (TK), and L1-L5 lumbar lordosis (LL).

As described in Aubin et al. [13], the SRS-Lenke-Aubin 3D classification incorporates two classifier-based transverse-plane modifiers—ORPD and AVR—and one complementary spatial descriptor, DAEVL. ORPD is computed as the angle between the sagittal plane and the plane defined by the apex and end vertebrae projected onto the transverse plane, and is stratified into three categories reflecting regional geometric transitions: mild (ORPD-1, < 70°), moderate (ORPD-2; 70–90°), and severe (ORPD-3; > 90°). AVR, defined as apical vertebral rotation relative to the same pelvis-anchored sagittal plane, is categorized into slight (s; < 10°), moderate (m; 10–20°), and large (l; > 20°). DAEVL, although not used as a modifier, quantifies the shortest perpendicular distance from the apex to the end-vertebrae line. It can be interpreted as a 3D version of the apical vertebral translation (AVT) [19], often used to guide surgical strategy, and complements ORPD and AVR by capturing the magnitude of apical displacement and regional convexity. Together, these operational thresholds provide a standardized quantitative basis for interpreting 3D deformity patterns within and across Lenke 1 subtypes.

### Statistical analyses

To explore the complementarity of the 2D and 3D descriptors, statistical analyses were conducted using RStudio (Posit Software, PBC, Boston, MA, USA; v. 2025.09.1 + 401). Normality of distributions was assessed using a combination of Shapiro–Wilk tests and skewness/kurtosis indicators, acknowledging the sensitivity of Shapiro–Wilk in large samples. Most variables showed acceptable skewness and kurtosis (± 2 and ± 7, respectively; [20]), with deviations primarily driven by a limited number of extreme values. To minimize the influence of such outliers, values exceeding ± 3.29 SD were winsorized by replacing them with the nearest admissible value at the ± 3.29 SD threshold, thereby limiting undue influence while preserving the overall structure of the dataset [21]. Following winsorization, all variables met normality assumptions, supporting the use of parametric analyses.

Pearson correlation coefficients (r) were computed to quantify the strength and direction of associations between the 2D and 3D descriptors across PT, MT, and TL/L regions. Interpretative thresholds followed Cohen’s guidelines: r < 0.1 (negligible), 0.1–0.3 (weak), 0.3–0.5 (moderate), 0.5–0.7 (moderate to elevated), and > 0.7 (strong) [22]. Statistical significance was set at p < 0.05, with highly significant findings reported as p < 0.001. Holm–Bonferroni correction was applied to adjust for multiple comparisons [23].

Analyses were structured to examine: (1) whether ORPD and AVR were interrelated within PT, MT, and TL/L regions; (2) whether coronal deformity (Cobb angle) was associated with ORPD and AVR; (3) whether sagittal alignment influenced transverse-plane orientation, specifically TK with MT ORPD and LL with TL/L ORPD; and (4) whether DAEVL correlated with Cobb angle, ORPD, and AVR, providing insight into the spatial expression of 3D curve morphology. These comparisons were systematically performed across PT, MT, and TL/L regions for all four analyses. An additional analysis examining the relationship between DAEVL and absolute AVT in the MT and TL/L regions was also conducted.

To evaluate the degree of overlap and complementarity between traditional 2D Lenke modifiers (lumbar A/B/C and thoracic −/N/+) and their 3D transverse-plane counterparts (ORPD modifiers in PT, MT, and TL/L), contingency tables were constructed and chi-square (χ^2^) tests of independence were applied. When expected cell counts fell below recommended thresholds, Fisher–Freeman–Halton exact tests were used instead. Cramér’s V quantified the strength of association, enabling assessment of whether 3D modifiers capture overlapping or distinct information relative to classical Lenke modifiers.

Given the dual geometric nature of ORPD—reflecting both coronal deviation and sagittal curvature—multiple linear regression models were constructed to evaluate the independent contributions of these components. For MT, Cobb angle and TK were tested as predictors of ORPD; for TL/L, Cobb angle and LL were examined. Diagnostic measures confirmed the absence of problematic multicollinearity (pairwise r < 0.8, variance inflation factors < 5, conditioning index < 30), ensuring the robustness of the regression estimates [24–26].

### Subtype-specific analyses: Lenke 1A, 1B, 1C

To evaluate heterogeneity within Lenke 1 curves, patients were subclassified as Lenke 1A (n = 104), 1B (n = 35), and 1C (n = 23). The full correlation structure was repeated for each subgroup, allowing comparisons of 3D–2D coupling across PT, MT, and TL/L. These subgroup analyses were considered exploratory, particularly for Lenke 1C given that the available sample size for Lenke 1C affords adequate statistical power mainly for detecting moderate-to-strong associations (r ≥ 0.55).

## Results

### Coronal vs transverse-plane relationships

**Fig. 2:**
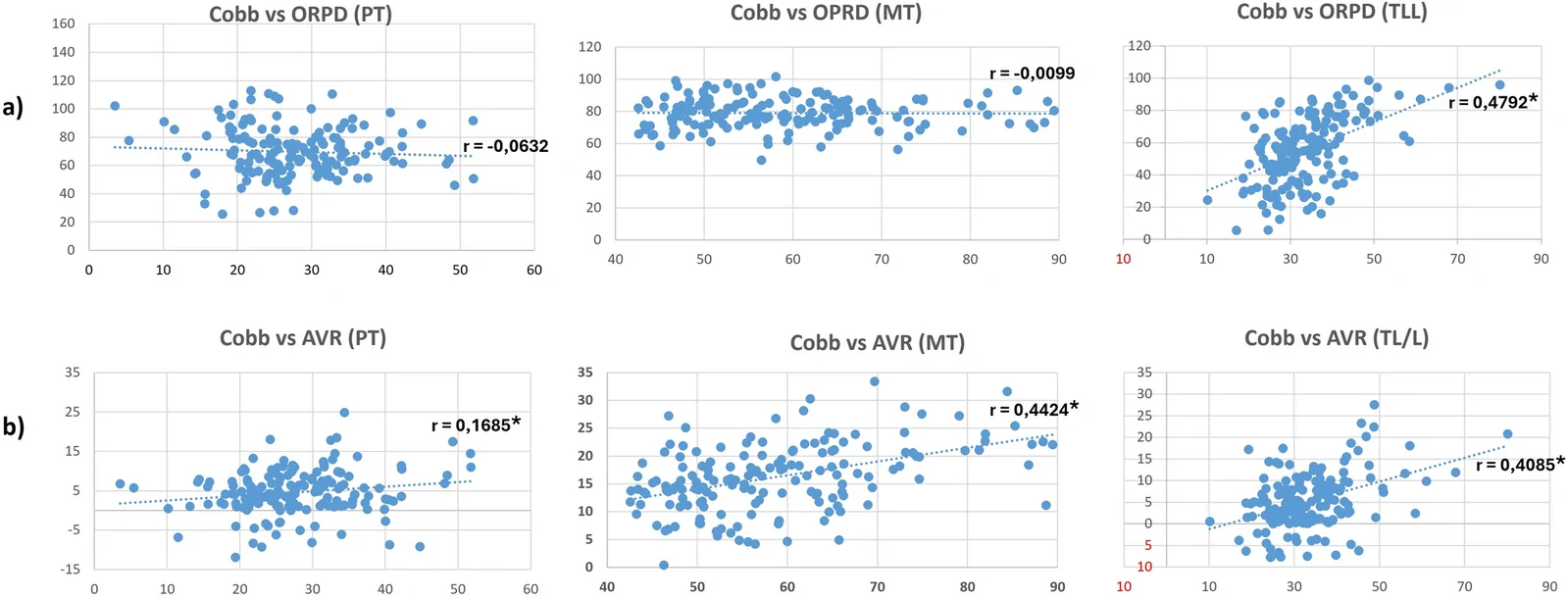
Scatter plots illustrating the relationships between Cobb angles (horizontal axis) and transverse-plane descriptors (vertical axis) across PT, MT, and TL/L regions. Panel **a** presents Cobb vs ORPD, and panel **b** Cobb vs AVR. Dotted lines represent linear regression fits. Reported r values are shown, with statistically significant correlations (p < 0.05) indicated by *

Across the cohort of 162 Lenke 1 patients, correlations between ORPD and Cobb magnitude demonstrated region-dependent behavior (Table 1; Fig. 2a). In the PT (r = −0.0632; p = 0.4240) and MT (r = −0.0099; p = 0.9002) regions, ORPD exhibited negligible and statistically non-significant associations with Cobb magnitude, indicating that coronal angular severity does not reliably predict the orientation of the regional plane of deformation in the thoracic spine. In contrast, the association was moderate in TL/L (r = 0.4792; p < 0.001), suggesting stronger geometric coupling in the compensatory lumbar region.

**Table 1:** Region-specific correlation matrix for the 162 Lenke 1 cases, reporting Pearson correlation coefficients (r) in bold, with corresponding p-values shown below each coefficient

All Lenke 1 cases	ORPD (PT, MT or TL/L)	AVR (PT, MT or TL/L)	DAEVL (PT, MT or TL/L)	TK	LL
Cobb PT	**−0.0632** 0.4240	**0.1685** 0.0317	0.5135* < 0.001	0.1702* 0.0304	
Cobb MT	**−0.0099** 0.9002	0.4424* < 0.001	0.4679* < 0.001	**0.0357** 0.6523	
Cobb TL/L	0.4792* < 0.001	0.4085* < 0.001	0.3415* < 0.001		**0.0972** 0.2184
ORPD PT		**0.1310** 0.0965	−0.2287* 0.0034	**−0.1101** 0.1631	
ORPD MT		−0.2177* 0.0054	0.2191* 0.005	−0.6344* < 0.001	
ORPD TL/L		0.5342* < 0.001	**0.1450** 0.0656		**0.1177** 0.1357
AVR PT	**−0.1317** 0.0965		**0.1601** 0.0418	**0.0727** 0.3581	
AVR MT	−0.2177* 0.0054		0.2342* 0.0027	**−0.004** 0.9591	
AVR TL/L	0.5342* < 0.001		0.4052* < 0.001		0.3196* < 0.001
DAEVL PT	−0.2294* 0.0034	**0.1601** 0.0418		**0.0688** 0.3843	
DAEVL MT	0.2191* 0.005	0.2342* 0.0027		−0.3503* < 0.001	
DAEVL TL/L	**0.1450** 0.0656	0.4052* < 0.001			0.4632* < 0.001

For each column (ORPD, AVR, DAEVL), correlations are presented with region-appropriate 2D parameters across PT, MT, and TL/L segments, consistent with the anatomical logic of the SRS-Lenke-Aubin 3D classification. Statistical significance (indicated with *) was assessed at p < 0.05, with Holm–Bonferroni correction applied to confirm robustness of significant findings

Cobb–AVR relationships followed a similar pattern but with generally higher effect sizes: weak correlations in PT (r = 0.1688; p = 0.0317) and moderate correlations in both MT (r = 0.4424; p < 0.001) and TL/L (r = 0.4085; p < 0.001) (Table 1; Fig. 2b). These results indicate that axial rotation increases with coronal magnitude, particularly at and below the thoracic apex.

### Independence between ORPD and AVR

**Fig. 3:**
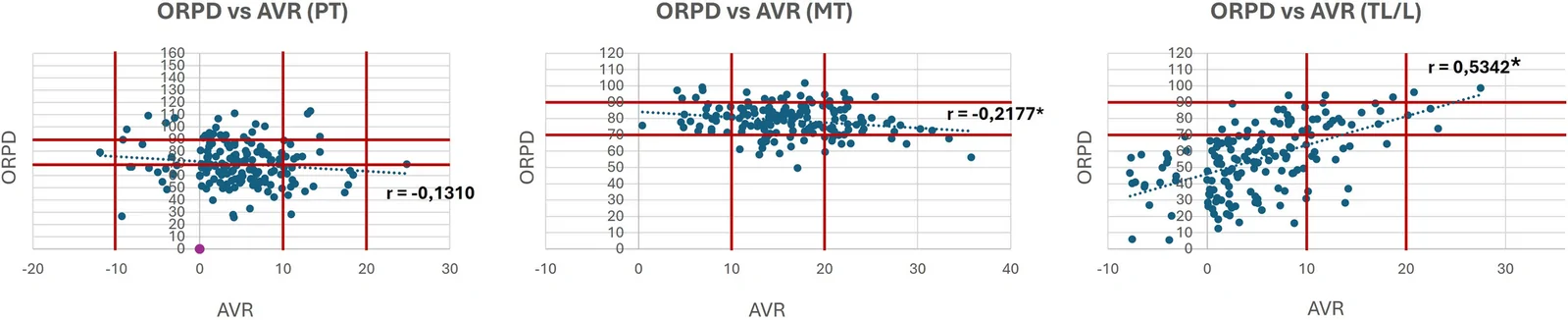
Distribution of transverse-plane deformity patterns across PT, MT, and TL/L regions. Each point represents an individual case plotted according to ORPD and AVR subclass combinations. Red lines indicate ORPD thresholds (70°, 90°) and AVR thresholds (10°, 20°), defining the subclass grid (1–3 and s–m–l). Reported r values are shown, with statistically significant correlations (p < 0.05) indicated by *

In the thoracic spine, ORPD and AVR showed no significant association in PT (r = −0.1310; p = 0.0965) and only a weak, though statistically significant, inverse association in MT (r = −0.2177; p = 0.0054). In contrast, their association was moderate to elevated in TL/L (r = 0.5342; p < 0.001) (Table 1; Fig. 3), reflecting more coherent coupling between plane orientation and apical rotation in the lumbar compensatory region.

### Sagittal alignment vs transverse-plane orientation

**Fig. 4:**
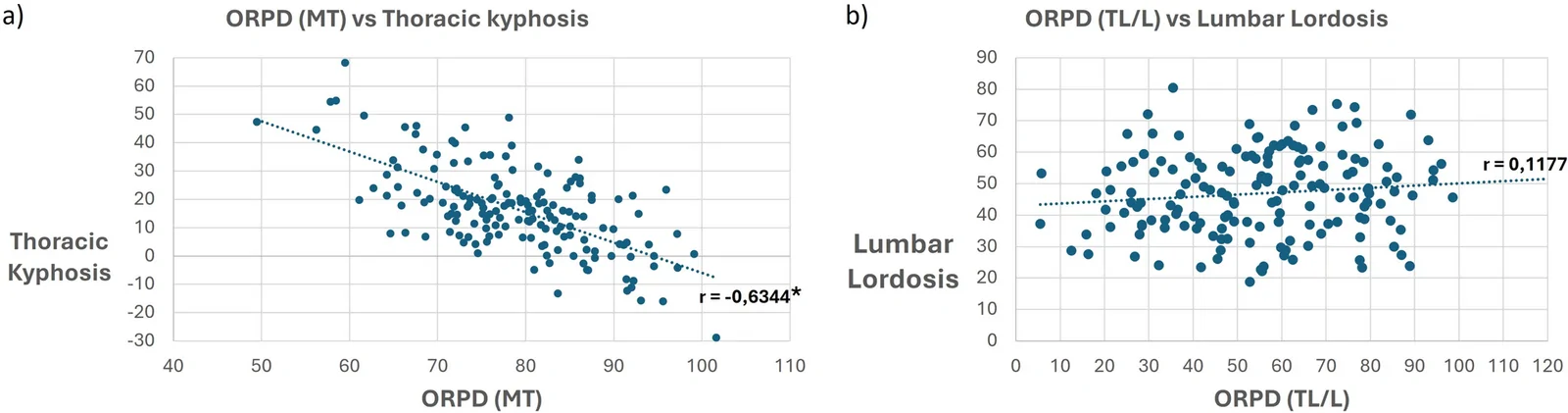
Relationships between sagittal alignment and transverse-plane orientation. **a** MT ORPD versus thoracic kyphosis (TK); **b** TL/L ORPD versus lumbar lordosis (LL). Dotted lines represent linear regression fits. Reported r values are shown, with statistically significant correlations (p < 0.05) indicated by *

TK showed a moderate-to-strong inverse correlation with MT ORPD (r = −0.6344; p < 0.001), confirming that decreasing TK (hypokyphosis) corresponds to increasing deviation of the thoracic deformation plane (Table 1; Fig. 4a). LL demonstrated no meaningful association with TL/L ORPD (r = 0.1177; p = 0.1357), indicating that lumbar sagittal curvature does not meaningfully relate to TL/L regional plane orientation in Lenke 1 curves (Table 1; Fig. 4b).

### Spatial descriptor (DAEVL) relationships

**Fig. 5:**
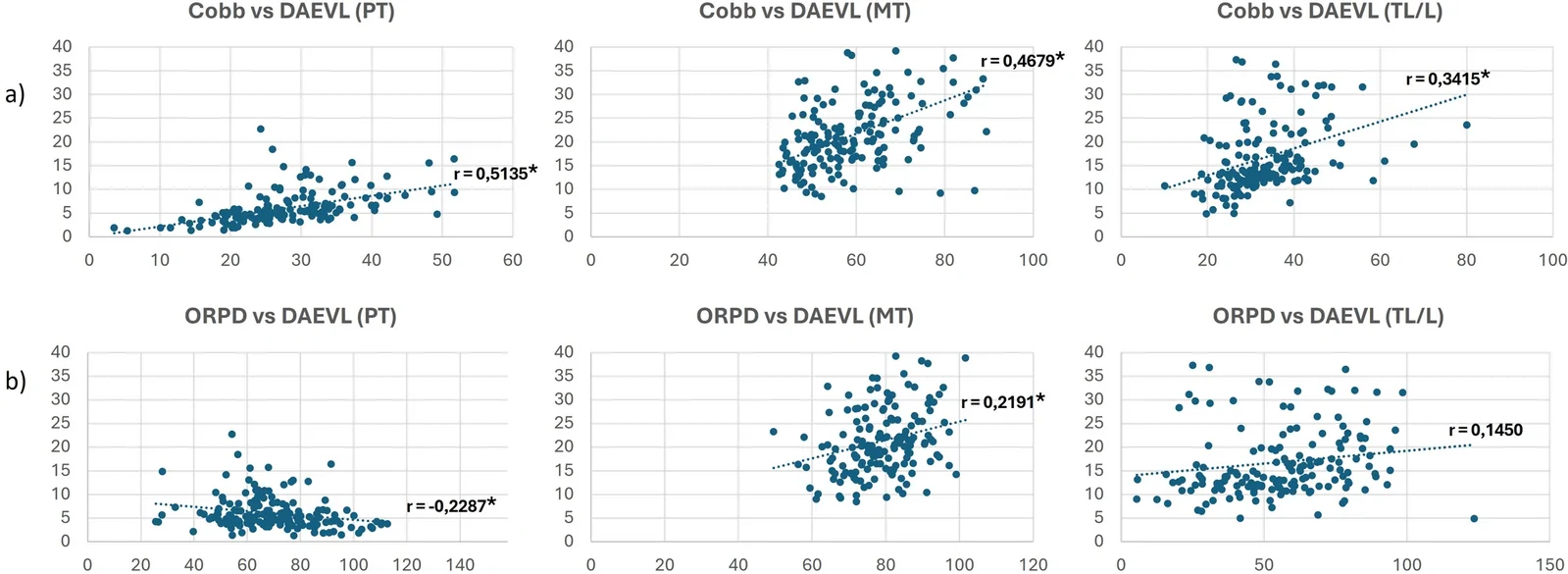
Relationships between apical displacement (DAEVL) and **a** Cobb angle, and **b** ORPD across PT, MT, and TL/L regions. Panel **a** shows moderate associations between coronal magnitude and apical displacement. Panel **b** illustrates weak-to-moderate associations between ORPD and DAEVL. Dotted lines represent linear regression fits. Reported r values are shown, with statistically significant correlations (p < 0.05) indicated by *

DAEVL exhibited moderate associations with Cobb magnitude across all regions (PT: r = 0.5135; MT: r = 0.4679; TL/L: r = 0.3415; all p < 0.001), reflecting that greater coronal severity is accompanied by increased apical displacement in the regional planes of deformity (Table 1; Fig. 5a). Correlations between DAEVL and ORPD were weak and statistically significant in PT (r = −0.2287; p = 0.0034) and MT (r = 0.2191; p = 0.0051), but not in TL/L (r = 0.1450; p = 0.0656). DAEVL–AVR correlations were weak in MT (r = 0.2342; p = 0.0027) and moderate in TL/L (r = 0.4052; p < 0.001) (Table 1).

**Fig. 6:**
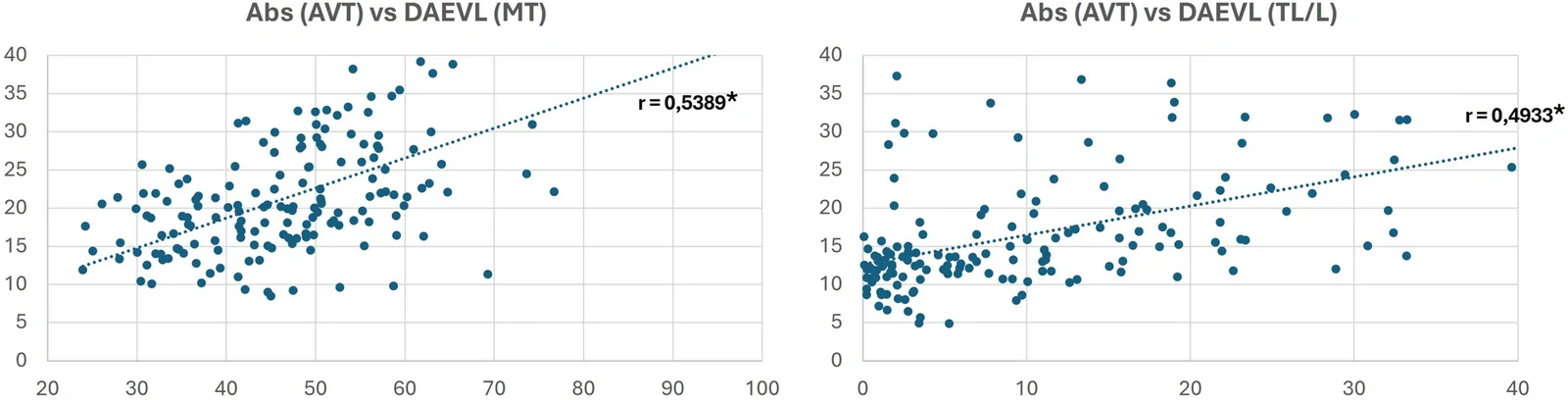
Scatterplots illustrating the relationship between absolute apical vertebral translation (AVT) and the 3D distance from the apex to the end-vertebrae line (DAEVL) in the MT (left) and TL/L (right) regions. Both AVT and DAEVL are expressed in millimeters (mm). Dotted lines represent linear regression fits. Reported r values are shown, with statistically significant correlations (p < 0.05) indicated by *

Additional analyses revealed moderate correlations between absolute apical vertebral translation (AVT) and apical displacement (DAEVL) in the MT (r = 0.5389, p < 0.001) and TL/L (r = 0.4933, p < 0.001) regions, indicating a consistent association between global coronal translation and regional spatial apex displacement across regions (Fig. 6).

### Associations between classical Lenke modifiers and ORPD-based transverse-plane modifiers

Contingency analyses (Table 2A) demonstrated a strong association between Lenke’s lumbar modifier (A/B/C) and the TL/L ORPD modifier (χ^2^ = 73.85, df = 4, p < 0.001; Cramér’s V = 0.48). Because one expected cell count was below 1, Fisher–Freeman–Halton exact testing was also performed and confirmed a highly significant association (p < 0.001). Examination of observed versus expected counts revealed marked deviations in lumbar C curves, particularly an underrepresentation in TL/L ORPD1 (observed 4 vs expected 16.90) and an overrepresentation in ORPD3 (observed 6 vs expected 0.99), indicating that TL/L ORPD captures aspects of lumbar involvement not fully reflected by the coronal lumbar modifier alone.

**Table 2:** Contingency tables (observed/expected): a) Lenke lumbar modifier vs TL/L ORPD modifier; b) Thoracic sagittal profile modifier vs MT ORPD modifier

a)				
Lumbar spine modifier	TL/L ORPD 1	TL/L ORPD 2	TL/L ORPD 3	Total
A	95 (76.40)	8 (23.11)	1 (4.49)	104
B	20 (25.71)	15 (7.78)	0 (1.51)	35
C	4 (16.90)	13 (5.11)	6 (0.99)	23
Total	119	36	7	162

b)				
Thoracic sagittal profile modifier	MT ORPD 1	MT ORPD 2	MT ORPD 3	Total
−	3 (8.00)	32 (38.33)	19 (7.67)	54
N	12 (14.07)	79 (67.44)	4 (13.49)	95
+	9 (1.93)	4 (9.23)	0 (1.85)	13
Total	24	115	23	162

Similarly, Lenke’s thoracic sagittal profile modifier (−/N/+) was strongly associated with the MT ORPD modifier (χ^2^ = 60.68, df = 4, p < 0.001; Cramér’s V = 0.43) (Table 2B). Because one expected cell count was below 1, Fisher–Freeman–Halton exact testing again confirmed a highly significant association (p < 0.001). Deviations between observed and expected counts were noted for specific MT combinations, including an overrepresentation of hyperkyphotic (+) profiles in ORPD1 (observed 9 vs expected 1.93) and of hypokyphotic (−) profiles in ORPD3 (observed 19 vs expected 7.67), indicating partial overlap but non-equivalence between sagittal profiling and transverse-plane ORPD characterization.

### Multivariate regression analyses of ORPD

Multivariate linear regression analyses confirmed region-specific contributions of coronal and sagittal parameters to ORPD. In the MT region, the model including Cobb MT and TK explained 39.5% of the variance in ORPD (adjusted R^2^ = 0.395; F(2,159) = 53.60, p < 0.001). Within this model, TK was the only independent predictor of ORPD MT (β = −0.376, p < 0.001), indicating that decreasing kyphosis was associated with increased transverse-plane deviation. In contrast, Cobb MT was not independently associated with ORPD MT (β = 0.011, p = 0.836).

In the TL/L region, the model including Cobb TL/L and LL explained 22.5% of the variance (adjusted R^2^ = 0.225; F(2,159) = 24.39, p < 0.001). In this region, Cobb TL/L was the sole independent predictor of ORPD TL/L (β = 1.049, p < 0.001), whereas LL was not independently associated with ORPD TL/L (β = 0.118, p = 0.304).

Diagnostic assessments confirmed the robustness of the regression estimates, with negligible correlations between coronal and sagittal predictors—specifically between MT Cobb and TK (r = 0.0357) and between TL/L Cobb and LL (r = 0.0972)—and no evidence of multicollinearity.

### Subtype analyses (Lenke 1A, 1B, and 1 C)

A progression in 3D behavior was observed across Lenke 1 subtypes, although subgroup-specific findings should be interpreted cautiously because of variable sample size.

Lenke 1A curves were characterized by relatively homogeneous 3D behavior, with mostly weak associations (Table 3). Notable exceptions included a strong inverse relationship between MT ORPD and TK (r = −0.7112; p < 0.001). At the TL/L level, associations between coronal magnitude and transverse-plane descriptors remained limited, with no significant relationship between Cobb and ORPD (r = 0.1441; p = 0.1444), and no significant associations between Cobb and DAEVL or between ORPD and AVR after Holm-Bonferroni correction.

**Table 3:** Correlation matrix for the Lenke 1A subgroup (n = 104)

Lenke 1A	ORPD (PT, MT or TL/L)	AVR (PT, MT or TL/L)	DAEVL (PT, MT or TL/L)	TK	LL
Cobb PT	**0.0296** 0.7651	**0.1167** 0.2381	0.4935* < 0.001	0.2181* 0.0261	
Cobb MT	**−0.1465** 0.1378	0.5027* < 0.001	0.4544* < 0.001	**0.0475** 0.6317	
Cobb TL/L	**0.1441** 0.1444	**0.0160** 0.8716	**0.2218** 0.0236		**−0.0853** 0.3890
ORPD PT		**−0.1726** 0.0797	**−0.1819** 0.0646	**−0.1863** 0.0582	
ORPD MT		**−0.2429** 0.0130	0.3077* 0.0015	−0.7112* < 0.001	
ORPD TL/L		**0.2612** 0.0074	**−0.1215** 0.2193		**0.0106** 0.9153
AVR PT	**−0.1726** 0.0797		0.1942* 0.0483	**0.0980** 0.3224	
AVR MT	**−0.2429** 0.0130		**0.1165** 0.2388	**0.0184** 0.8528	
AVR TL/L	**0.2612** 0.0074		**0.0696** 0.4829		**0.1824** 0.0639
DAEVL PT	**−0.1819** 0.0646	0.1942* 0.0483		**0.0574** 0.5628	
DAEVL MT	0.3077* 0.0015	**0.1165** 0.2388		−0.3992* < 0.001	
DAEVL TL/L	**−0.1215** 0.2193	**0.0696** 0.4829			0.4976* < 0.001

Pearson correlation coefficients (r) in bold and corresponding p-values shown below are reported for ORPD, AVR, and DAEVL relative to DAEVL relative to regional Cobb angles, thoracic kyphosis (TK), and lumbar lordosis (LL) across PT, MT, and TL/L segments (*Indicates p < 0.05)

Lenke 1B curves exhibited greater variability compared with 1 A, with a larger number of statistically significant associations overall, reflecting a transitional 3D behavior that is intermediate in nature but not necessarily consistent across descriptors and regions (Table 4). Several moderate associations were observed, including an inverse association between ORPD MT and TK (r = −0.5635; p < 0.001), as well as a positive association between Cobb TL/L and ORPD TL/L (r = 0.5088; p = 0.0018). At the MT level, AVR MT and DAEVL MT were modestly associated (r = 0.3637; p = 0.0317), whereas several other relationships remained weak and inconsistent, underscoring the heterogeneous and intermediate behavior of the 1B subtype rather than a clearly defined coupling pattern.

**Table 4:** Correlation matrix for the Lenke 1B subgroup (n = 35)

Lenke 1B	ORPD (PT, MT or TL/L)	AVR (PT, MT or TL/L)	DAEVL (PT, MT or TL/L)	TK	LL
Cobb PT	**−0.1988** 0.2521	**0.2751** 0.1098	0.6373* < 0.001	**0.1537** 0.3779	
Cobb MT	**0.0683** 0.6965	**0.2538** 0.1412	**0.2193** 0.2056	**0.1868** 0.2826	
Cobb TL/L	0.5088* 0.0018	**0.0903** 0.6059			**−0.0731** 0.6765
ORPD PT		**−0.0118** 0.9464	**−0.0517** 0.7680	**−0.1279** 0.4642	
ORPD MT		**−0.2087** 0.2289	**0.0328** 0.8516	−0.5635* < 0.001	
ORPD TL/L		**0.2268** 0.1901	**−0.3452** 0.0423		**−0.3890** 0.0209
AVR PT	**−0.0118** 0.9464		**0.2258** 0.1921	**−0.0251** 0.8864	
AVR MT	**−0.2087** 0.2289		0.3637* 0.0317	**0.0854** 0.6257	
AVR TL/L	**0.2268** 0.1901		**0.3119** 0.0681		**0.3031** 0.0767
DAEVL PT	**−0.2934** 0.0872	**0.2258** 0.1921		**0.2394** 0.1660	
DAEVL MT	**0.0328** 0.8516	0.3637* 0.0317		**−0.2524** 0.1435	
DAEVL TL/L	**−0.3452** 0.0423	**0.3119** 0.0681			**0.1850** 0.2873

Pearson correlation coefficients (r) in bold and corresponding p-values shown below are presented for ORPD, AVR, and DAEVL relative to regional Cobb angles, thoracic kyphosis (TK), and lumbar lordosis (LL) across PT, MT, and TL/L segments. This table highlights the variability and intermediate 3D coupling patterns observed in this subgroup. (*Indicates p < 0.05)

Lenke 1C curves demonstrated more pronounced deformity patterns at both MT and TL/L levels but remained statistically fragile because of limited sample size (Table 5). Within the structural thoracic segment, Cobb MT was significantly associated only with DAEVL MT (r = 0.7102; p < 0.001), whereas associations with ORPD MT and AVR MT did not remain correlated after Holm-Bonferroni correction. At the TL/L level, ORPD TL/L showed a moderate association with AVR TL/L (r = 0.561; p = 0.005), while the association between Cobb TL/L and ORPD TL/L did not remain significant after Holm-Bonferonni correction. ORPD MT was inversely associated with TK (r = −0.4894; p = 0.0178). Because the 1C subgroup remains underpowered for detecting weak-to-moderate correlations, the absence of significance for some associations should not be interpreted as evidence of independence. Rather, these findings should be interpreted cautiously and considered exploratory and hypothesis-generating.

**Table 5:** Correlation matrix for the Lenke 1C subgroup (n = 23)

Lenke 1C	ORPD (PT, MT or TL/L)	AVR (PT, MT or TL/L)	DAEVL (PT, MT or TL/L)	TK	LL
Cobb PT	**−0.1289** 0.5579	**0.5722** 0.0043	0.6521* < 0.001	**0.1835** 0.4021	
Cobb MT	**0.4261** 0.0426	**0.4377** 0.0367	0.7102* < 0.001	**−0.2291** 0.2931	
Cobb TL/L	**0.4259** 0.0427	**0.2194** 0.3145	**0.0401** 0.8560		**−0.0523** 0.8127
ORPD PT		**−0.3287** 0.1256	**−0.2060** 0.3457	**0.1667** 0.4470	
ORPD MT		**−0.1133** 0.6068	**0.4437** 0.0339	−0.4894* 0.0178	
ORPD TL/L		**0.5610** 0.0054	0.4437* 0.0339		**−0.1563** 0.4765
AVR PT	**−0.3287** 0.1256		**0.2628** 0.2257	**0.0064** 0.9771	
AVR MT	**−0.1133** 0.6068		**0.3655** 0.0864	**0.0168** 0.9392	
AVR TL/L	**0.5610** 0.0054		**0.3894** 0.0663		**0.1946** 0.3736
DAEVL PT	**−0.2060** 0.3457	**0.2628** 0.2257		**0.3310** 0.1228	
DAEVL MT	**0.4437** 0.0339	**0.3655** 0.0864		**−0.3634** 0.0883	
DAEVL TL/L	**−0.2367** 0.2768	**0.3894** 0.0663			**0.2695** 0.2137

Pearson correlation coefficients (r) in bold and corresponding p-values shown below are reported for ORPD, AVR, and DAEVL relative to regional Cobb angles, thoracic kyphosis (TK), and lumbar lordosis (LL) across PT, MT, and TL/L segments. Given the sample size, findings should be interpreted cautiously, as moderate associations may not reach statistical significance. (*Indicates p < 0.05)

## Discussion

The present Lenke 1–specific analysis provides refined insight into the regional behavior of transverse-plane deformities within the SRS-Lenke-Aubin 3D classification framework and suggests that ORPD, AVR, and DAEVL provide additional, complementary information that is only partially captured by conventional 2D radiographic measures. Building on full-cohort analyses, these results show that the behavior of these descriptors varies meaningfully between structural and compensatory regions. Importantly, this refined 3D characterization is consistent with earlier observations of heterogeneous thoracic 3D phenotypes within Lenke 1 curves [4].

These findings should be interpreted in the context of prior work on the SRS–Lenke–Aubin 3D classification [13] and subsequent full-cohort analyses across Lenke types [14]. While the present results are conceptually consistent with the established three-dimensional nature of AIS, they provide new subtype-specific insights that are not directly inferable from prior analyses. In particular, this study highlights differential regional coupling patterns within Lenke 1 curves, the distinct contributions of sagittal and coronal parameters to MT and TL/L ORPD, and the presence of a graded 3D progression across Lenke 1A–1C subtypes. These findings therefore extend previous work by providing quantitative and clinically interpretable refinement at the subtype level rather than constituting a simple re-analysis of existing data.

### Distinct transverse-plane behavior in structural versus compensatory regions

A central finding is the contrast between thoracic and lumbar regions in how transverse-plane deformity relates to coronal and sagittal parameters. In the MT region—the structural driver of Lenke 1 curves—correlations between Cobb angle and ORPD were negligible and statistically non-significant, and those with AVR were only moderate. This indicates that thoracic coronal severity does not predict the orientation of the deformation plane and only partially reflects axial rotation. Such geometric decoupling is consistent with the known complexity and heterogeneity of thoracic scoliosis, in which rib-cage constraints, segmental stiffness, and patient-specific anatomy give rise to diverse 3D configurations and underpin the wide variety of clinical situations routinely encountered by pediatric spine surgeons in daily practice.

Even the strongest observed associations were moderate and explain only a limited proportion of the variance, indicating substantial independence between 2D and 3D descriptors. In the TL/L region—by definition a nonstructural, compensatory curve in Lenke 1 patterns—associations among Cobb magnitude, ORPD, AVR, and DAEVL were somewhat slightly stronger, though still moderate, suggesting a more coherent compensatory 3D adjustment pattern in which coronal deviation, axial rotation, and spatial apex displacement tend to evolve together.

Importantly, several associations observed in this study fall within the moderate range (r ≈ 0.3–0.55), corresponding to approximately 9–30% shared variance (r^2^). Within the framework of this study, these effect sizes are interpreted as partial associations rather than strong coupling, consistent with the objective of assessing complementarity rather than dependence between 2D and 3D descriptors. The predominance of weak-to-moderate correlations—and the absence of consistently strong relationships—therefore supports the non-redundant, complementary nature of transverse-plane descriptors. In the overall cohort (n = 162), the study is sufficiently powered to detect small-to-moderate associations, indicating that these findings reflect true partial independence rather than insufficient statistical power. In contrast, for smaller subgroups—particularly Lenke 1C (n = 23)—only relatively strong correlations (r ≳ 0.5–0.55) can be reliably detected, and moderate associations may remain undetected. Accordingly, non-significant or weak-to-moderate findings in these subgroups should be interpreted with caution and considered exploratory, while also remaining consistent with the conceptual objective of demonstrating complementarity.

### Complementarity of ORPD and AVR in thoracic curves

Consistent with the companion study on transverse-plane descriptor complementarity [14], ORPD and AVR capture largely independent yet synergistic aspects of thoracic deformity, with ORPD primarily reflecting regional plane orientation and AVR quantifying local rotational severity. This complementarity helps explain why severe plane rotation may coexist with only moderate apical rotation in certain Lenke 1 patterns, including those with pronounced hypokyphosis.

The weak or negligible correlations between ORPD and AVR in PT and MT regions confirm that these descriptors capture distinct geometric dimensions. ORPD reflects the global orientation of the regional deformation plane, whereas AVR quantifies local axial torsion at the apex. Their independence in thoracic regions supports their combined use in characterizing structural curves, where deformity patterns are highly variable and cannot be reduced to a single transverse-plane parameter. In contrast, the moderate coupling observed in TL/L suggests that plane orientation and rotation become more interrelated in compensatory regions, while still remaining non-redundant.

### Sagittal alignment and transverse-plane morphology

The inverse association between TK and MT ORPD indicates that hypokyphosis is associated with increased transverse-plane deviation of the thoracic curve. Although this relationship is moderate to strong, it does not allow inference of transverse-plane geometry from sagittal alignment alone. However, interpretation of these findings should consider that spinal regions are defined differently across planes: sagittal alignment parameters are computed over fixed anatomical ranges (T5–T12 for TK and L1–L5 for LL), whereas coronal curves and 3D descriptors rely on patient-specific end vertebrae defined by Lenke guidelines. As these boundaries differ on average by approximately 1–1.5 vertebral levels, sagittal parameters and transverse-plane descriptors may not always reflect identical spinal segments, which may partly explain some less intuitive ORPD modifiers when interpreted alongside conventional sagittal curves.

The absence of a meaningful association between LL and TL/L ORPD further underscores the region-specific and non-uniform nature of sagittal–transverse interactions and suggests that future analyses may benefit from defining spinal regions tridimensionally rather than solely according to standard coronal criteria.

### Added value of DAEVL as a spatial descriptor

DAEVL showed moderate associations with Cobb magnitude across all regions, while its relationships with ORPD were weak and region-dependent. Correlations with AVR followed a similar pattern—weak in PT and structural MT, becoming moderate in compensatory TL/L. These findings indicate that transverse-plane apical displacement is only partially captured by regional plane orientation. Taken together, they support the role of DAEVL as a complementary descriptor that quantifies the regional and spatial excursion of the apex relative to the end vertebrae—information not conveyed by angular measures such as Cobb magnitude or by global-frame metrics such as AVT.

Interpretation of the relationships between AVT and DAEVL should consider that both metrics are dimensional and therefore influenced by patient size. Moreover, AVT is defined within a global coronal reference frame, whereas DAEVL is computed within a region-specific 3D plane aligned with the end-vertebrae axis. Despite these methodological differences, their moderate correlations across regions support a coherent link between global translational behavior and region-specific 3D apex displacement, while underscoring that AVT and DAEVL capture related yet non-interchangeable aspects of deformity.

### Interpretation of unexplained variance

A substantial proportion of the variance in ORPD remains unexplained by conventional 2D parameters. This likely reflects the influence of additional biomechanical and anatomical factors not captured in standard radiographic measures, including regional torsional stiffness, three-dimensional vertebral and disc wedging asymmetry, rib-cage morphology, pelvic alignment, and growth-related modulation of spinal deformity. In addition, patient-specific factors such as age and sex may contribute to inter-patient variability in transverse-plane descriptors, although these variables were not included as primary covariates in the present imaging-based analysis. This unexplained variance reinforces the rationale for retaining ORPD as a distinct descriptor rather than reducing it to a surrogate of Cobb magnitude or sagittal profile.

### Conceptual consistency and complementarity with Lenke modifiers

Inspection of observed versus expected counts revealed patterns that are largely consistent with the conceptual logic underlying both the Lenke framework and the SRS-Lenke-Aubin 3D classification, while also highlighting areas of partial divergence. As expected, Lenke lumbar modifier C was associated with a higher prevalence of severe transverse-plane orientation in the TL/L region (ORPD-3), whereas Lenke 1A curves were more frequently associated with mild TL/L transverse-plane orientation (ORPD-1). Similarly, the strong association observed between the thoracic sagittal profile modifier and MT ORPD aligns with biomechanical expectations: hypokyphotic curves (−) tended to be overrepresented in higher ORPD categories, whereas hyperkyphotic curves (+) were more frequently associated with milder MT ORPD orientations.

Taken together, these findings suggest that while the traditional Lenke modifiers capture part of the underlying 3D behavior, the ORPD-based transverse-plane modifiers provide additional and more granular information, reinforcing the rationale for integrating transverse-plane descriptors into an extended Lenke-based system. Rather than being redundant, the 3D descriptors refine and extend the Lenke framework by quantifying how coronal and sagittal characteristics translate—sometimes imperfectly—into three-dimensional regional deformation.

### Subtype-specific interpretation: Lenke 1A, 1B, and 1C

Subtype analyses revealed distinct 3D signatures across Lenke 1A, 1B, and 1C curves, refining interpretation beyond the global Lenke 1 profile. Overall, associations in the TL/L segment remained weak to moderate, with no descriptor pairs consistently exhibiting strong coupling. Nevertheless, a progressive and clinically meaningful gradient in 3D involvement emerged across subtypes.

Lenke 1A curves showed relatively homogeneous behavior with limited transverse-plane coupling, consistent with mild lumbar involvement. Lenke 1B curves exhibited the greatest variability, reflecting a transitional and incompletely defined morphologic pattern that occupies a gray zone between predominantly thoracic-driven and more lumbar-influenced 3D behaviors. In Lenke 1C curves, 3D involvement extended beyond the structural MT curve to include more pronounced and complex lumbar compensation. While Lenke 1A supports reliable detection of moderate associations, Lenke 1B and especially Lenke 1C require larger effect sizes for consistent detection. As a result, absence of association in these subgroups should not be interpreted as evidence of independence, but rather in light of statistical power limitations. Accordingly, findings in smaller subgroups are best considered exploratory and hypothesis-generating, and should be interpreted in conjunction with the broader conceptual objective of assessing complementarity between descriptors.

### Inter-patient variability and potential clinical implications

**Fig. 7:**
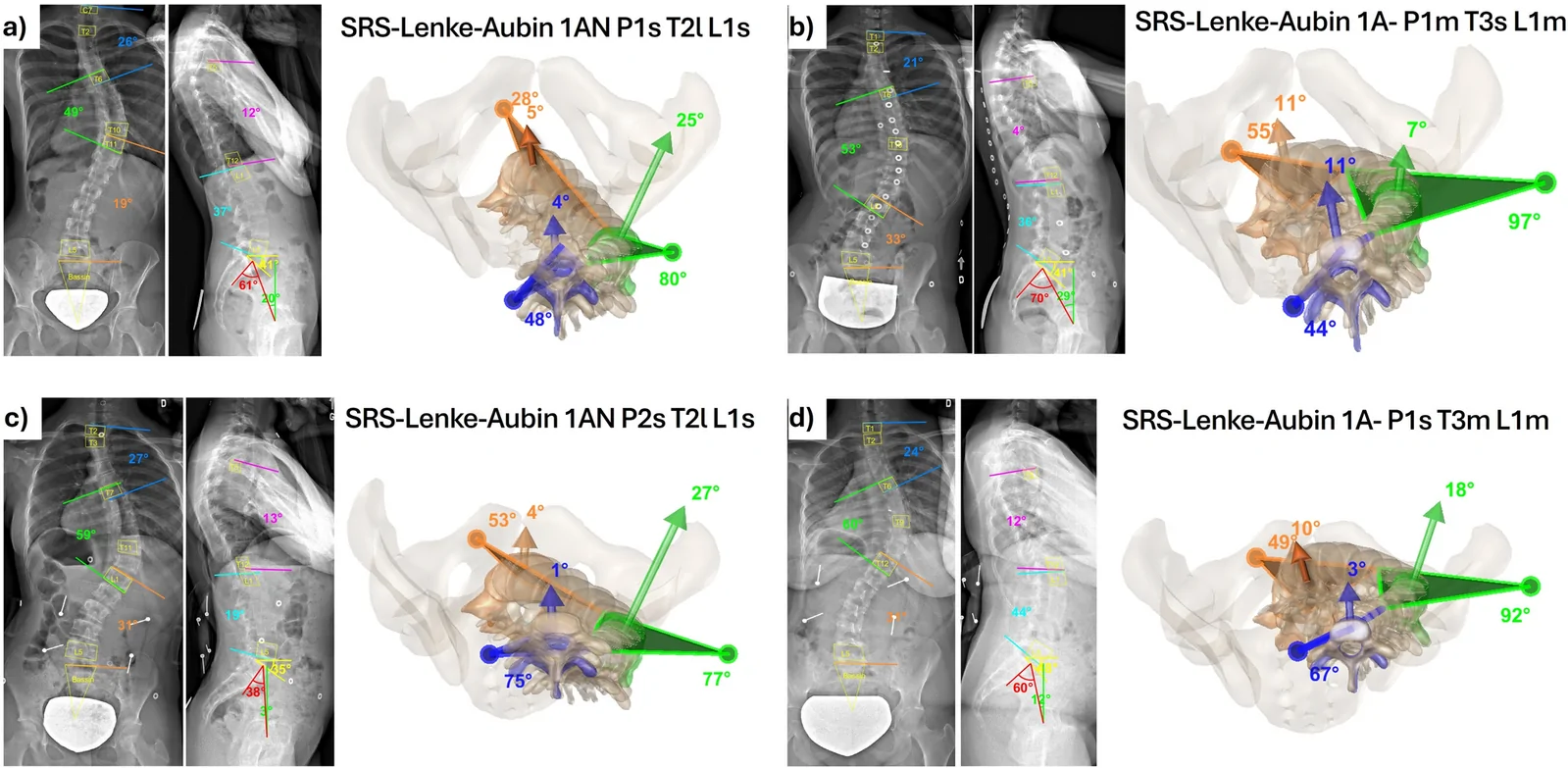
Representative Lenke 1A cases illustrating discrepancies between sagittal profile, thoracic coronal curve extent, and transverse-plane geometry. Panels **a** and **b** show two Lenke 1A patients with comparable MT Cobb angles but markedly different ORPD and AVR, including a case with low TK combined with preserved PT kyphosis (T2–T5) and pronounced MT ORPD (panel **b**; ORPD > 90°). Panels **c** and **d** present a second pair with similar MT Cobb angles but divergent transverse-plane morphology; panel **d** illustrates a patient with lordokyphosis (globally negative thoracic kyphosis) associated with inverted MT transverse-plane orientation (ORPD > 90°)

Beyond population-level associations, representative individual cases further illustrate the possible value of transverse-plane descriptors (Fig. 7). Several Lenke 1A patients exhibited marked discrepancy between sagittal profile, thoracic coronal curve extent, and transverse-plane geometry. This included cases with low TK combined with preserved PT kyphosis (T2–T5) or with lordokyphosis (negative TK), yet associated with pronounced main thoracic ORPD (> 90°) but different AVR. In addition, Lenke 1A curves with nearly identical MT Cobb angles demonstrated substantial variability in ORPD and AVR, underscoring that similar coronal severity may correspond to fundamentally different 3D morphologies and potentially distinct corrective considerations. These findings reinforce that coronal magnitude alone is insufficient to capture the three-dimensional diversity of thoracic deformities.

Beyond statistical associations, inspection of scatterplots revealed substantial inter-patient heterogeneity. For a given Lenke 1 Cobb angle, wide dispersion was observed in both ORPD and AVR, and similar transverse-plane descriptors were sometimes present across a broad range of coronal magnitudes. These findings reinforce that coronal magnitude alone is insufficient to capture the three-dimensional diversity of thoracic deformities and highlight the relevance of integrating transverse-plane descriptors into both research stratification and surgical interpretation.

Although the present study does not evaluate clinical outcomes or surgical decision-making directly, the identified transverse-plane descriptors provide a structured and interpretable framework to characterize three-dimensional deformity patterns that may inform surgical interpretation. In particular, they may contribute to a more nuanced appreciation of curve morphology, potentially supporting considerations related to correction strategies and fusion level selection, including LIV and UIV. However, these applications remain hypothetical at this stage, as predictive validity was not assessed in this descriptive study. Ongoing and planned investigations within the SRS 3D Classification Task Force aim to specifically address these clinically oriented questions and evaluate the extent to which transverse-plane descriptors may contribute to outcome prediction and surgical planning.

### Limitations

This study has limitations. First, although the overall sample was substantial, subgroup analyses—particularly for Lenke 1C—were comparatively small and should be considered exploratory. Second, the study is retrospective and descriptive, and no clinical outcomes or postoperative decision variables were analyzed. Third, while the 3D reconstruction workflow has been previously validated, all quantitative descriptors remain dependent on the fidelity of landmark identification and image reconstruction. Finally, although the distribution of points on the regression plots visually suggested predominantly linear relationships, nonlinear associations caused by sample heterogeneity cannot be excluded and may warrant exploration in future work.

## Conclusion

This Lenke 1–specific analysis suggests that the transverse-plane descriptors introduced in the SRS-Lenke-Aubin 3D classification—ORPD, AVR, and DAEVL—provide additional, complementary information that is not fully explained by conventional 2D radiographic measures. In Lenke 1 curves, these descriptors exhibit distinct and region-dependent behaviors, reflecting the intrinsic 3D complexity of this curve type.

In the structural thoracic region, ORPD and AVR remain largely independent, indicating that regional plane orientation and apical rotation describe complementary but distinct aspects of thoracic deformity that cannot be inferred from coronal magnitude alone. In contrast, the compensatory TL/L region shows more coherent—but still moderate—coupling among coronal severity, transverse-plane orientation, axial rotation, and apical displacement, consistent with a more constrained and biomechanically driven compensatory response. Across Lenke 1 subtypes, this framework further reveals distinct 3D signatures from 1A to 1C, with increasing variability and coordination of coronal, axial, and spatial descriptors.

Taken together, these Lenke 1–specific findings support the conceptual foundations of the SRS-Lenke-Aubin 3D classification. The combined use of ORPD, AVR, and DAEVL enhances the characterization of regional and subtype-specific deformity patterns and may support a more informed interpretation of thoracic curve behavior and compensatory mechanisms in Lenke 1 AIS. Future outcome-based studies are required to determine their clinical impact.

## Data Availability

In the spirit of open science, inquiries regarding potential use of the data for future research may be directed to the corresponding author. Access is subject to ongoing studies of the SRS 3D Classification Task Force currently making use of these datasets.
